# High SARS-CoV-2 attack rates in areas with low detection after community transmission established in Port Vila, Vanuatu, April 2022

**DOI:** 10.5365/wpsar.2024.15.1.1078

**Published:** 2024-02-22

**Authors:** Florita Toa, Wendy Williams, Chaturangi Yapa, Matthew Cornish, Melissa Binihi, Caroline van Gemert

**Affiliations:** a Vanuatu College of Nursing Education, Port Vila, Vanuatu.; b Vanuatu Ministry of Health, Port Vila, Vanuatu.; c Vanuatu Health Program, Port Vila, Vanuatu.; d Private physician, Port Vila, Vanuatu.; e Burnet Institute, Melbourne, Victoria, Australia.

## Abstract

**Objective:**

On 4 March 2022, the first community-acquired case of severe acute respiratory syndrome coronavirus 2 (SARS-CoV-2) was reported in Vanuatu, with community transmission occurring subsequently. It was expected that the number of notified SARS-CoV-2 cases would be an underestimate of the true infection rate of this outbreak; however, the magnitude of underreporting was unknown. The purpose of this study was to provide a population-based estimate of SARS-CoV-2 infection shortly after the first reports of community transmission, to understand the level of underdetection and undernotification in Vanuatu and thus to inform ongoing prevention and response activities.

**Methods:**

We conducted a cross-sectional SARS-CoV-2 prevalence study in two geographical administrative areas in Port Vila, Vanuatu in April 2022. All residents in selected areas were eligible. Trained teams conducted demographic and behavioural interviews and collected nasal specimens. Specimens were tested by polymerase chain reaction. The primary outcomes were the rates of SARS-CoV-2 attack (point prevalence) and cumulative attack, underdetection, notification and household secondary attack.

**Results:**

A total of 252 people from 84 households participated. Among 175 people who had a sample collected, 91 were SARS-CoV-2-positive (attack rate 52.0%). Most cases had not been detected before the study (underdetection rate 91.5%). More than half of previously detected cases were notified (notification rate 65.2%).

**Discussion:**

Within the first few weeks of community transmission, more than half of participants in the selected areas had evidence of SARS-CoV-2 infection; however, most infections had been undetected. This study provides important information about the rapid spread of novel infectious diseases in Vanuatu.

At the end of 2021, the Pacific island country of Vanuatu was one of about 10 countries globally that had not yet experienced community transmission of severe acute respiratory syndrome coronavirus 2 (SARS-CoV-2), the virus that causes coronavirus disease (COVID-19). (*1*) Between 2020 and 2021, Vanuatu (which comprises 83 islands and has a population of 302 000) implemented stringent and successful policies to prevent importation and community transmission of SARS-CoV-2, and only seven border cases were detected among over 8000 returning citizens until the end of 2021. (*2*, *3*)

The highly transmissible B.1.1.529 (Omicron) variant of SARS-CoV-2 was first identified globally in November 2021. (*4*) Between December 2021 and January 2022, Vanuatu paused all repatriation flights for returning citizens and residents. Repatriation flights resumed on 16 February 2022; from 17 February to 4 March, 39 cases were detected among travellers (*n* = 27) and front-line border workers (*n* = 12). (*5*)

On 4 March 2022, the first locally acquired case of SARS-CoV-2 was detected in the capital city, Port Vila, in a person who had not undertaken international travel. (*5*) This case was asymptomatic and detected through routine screening at Vila Central Hospital. An additional 13 community cases, all symptomatic, were subsequently identified after they presented to the hospital-based testing clinic on 5 March 2022, indicating community transmission. (*5*) The test positivity rate in Port Vila increased from 16% on 7 March to a peak of 52% on 26 March (data not publicly available, personal communication with the authors from the National Surveillance, Research & Emergency Response Unit [NSRERU]).

The Vanuatu Ministry of Health implemented several surveillance-strengthening activities between 2020 and 2021, including developing standard operating procedures (SOPs) for managing suspected and confirmed cases, training health-care workers on SOPs and implementing electronic notifications for new SARS-CoV-2 diagnoses. (*2*, *6*) However, gaps remained; for example, there was limited awareness of notification requirements among health-care workers. Owing to limited access to SARS-CoV-2 tests (antigen and polymerase chain reaction [PCR]) and an expected high number of infections due to high-density housing, it was anticipated that the number of notified SARS-CoV-2 cases would underestimate the true infection rate in Vanuatu during a community outbreak. However, the magnitude of this underreporting was unknown. The purpose of this study was to provide a population-based estimate of SARS-CoV-2 infection shortly after community transmission was first reported, to understand the level of underdetection and undernotification in Port Vila to inform ongoing prevention and response activities.

## Methods

### Study design

We conducted a cross-sectional SARS-CoV-2 prevalence study and triangulated data with notification data.

### Study setting

Two geographically defined administrative units in Port Vila, Vanuatu were purposively selected based on a population size of about 300 people and at least one confirmed case notified to the NSRERU by 25 March 2022. The administrative units were defined by the Vanuatu National Statistics Office, and the population of about 300 people was deemed to be a manageable sample size.

### Study population

The eligible population included all residents (defined as those whose main dwelling was in one of the administrative areas) who were at home in the two selected administrative areas at the time of fieldwork. A stay-at-home order was in effect during the study period; (*7*) therefore, it was expected that most residents would be at home. Where possible, residents who were not present during data collection were approached to participate by field research teams within 2–3 days of fieldwork. Unattended households were not included. Any nonresidents present during the study were not eligible to participate; nonresidents were identified by field teams asking, “Is this your usual place of residence?”

### Recruitment and consent

A three-stage process was used to invite eligible people to participate. The first stage was liaison and approvals with key local stakeholders, known locally as the municipality secretary and area administrator of the selected communities, and the second was through the village chief and community leaders. Finally, once approval for the study had been granted by the village chief and community leaders, community engagement teams went door-to-door to all households listed on the administrative maps to explain the study, address any concerns and obtain informed consent. Data collection teams then visited households to interview residents and collect nasopharyngeal samples.

### Data collection

Data were collected by trained interviewers, most of whom were health professionals or nursing students. The questionnaire collected demographic information, symptom history, health-care seeking behaviour and compliance with prevention measures. Demographic information included sex, age, country of nationality and household size. Symptoms experienced during the previous 2 weeks included cough, fever, headache, aches and pains, runny nose, sore throat, fatigue, loss of smell, nausea, shortness of breath, vomiting, diarrhoea or chest pain; a period of 2 weeks (rather than the 4 weeks since the beginning of the outbreak) was used to increase the accuracy of participant recall.

Health-care seeking behaviour included SARS-CoV-2 vaccination status and testing history. At the time of the study, public testing for SARS-CoV-2 was only available at a limited number of government-run testing clinics and at Vila Central Hospital. Some workplaces and individuals had privately procured point-of-care SARS-CoV-2 antigen tests; however, these tests were not widely available for purchase in Vanuatu. “Fully vaccinated” was defined as having received two doses of a COVID-19 vaccine that had received World Health Organization (WHO) emergency use listing as of 2 March 2022. (*8*) The two vaccines available in Vanuatu at this time were BBIBP-CorV (Sinopharm) and AstraZeneca, and 44% of the adult population was considered fully vaccinated on 23 January 2022. (*9*) Compliance with prevention measures such as mask use, staying home except for essential movements and practising hand hygiene was assessed using a three-point Likert scale (always, sometimes or never).

Trained nursing students collected SARS-CoV-2 nasopharyngeal swabs. Participants reporting a positive SARS-CoV-2 test within the previous 2 weeks chose whether to be retested; when a previous positive SARS-CoV-2 test was reported, this test was searched for in the national surveillance data set using a name, date of birth and address. Questionnaires were entered into a custom Google form and transferred to Microsoft Excel and Stata for analysis.

### Laboratory testing

Specimens collected for this study were transported to Vila Central Hospital in a temperature-controlled vaccine carrier box for laboratory testing. Specimens were tested using the GeneXpert SARS-CoV-2 assay, a reverse transcription PCR (RT–PCR) based assay for the detection of SARS-CoV-2. Meta-analyses have consistently reported high pooled sensitivity (> 98%) and pooled specificity (> 95%) for this assay. (*10*, *11*)

### Data analysis

The primary outcomes were rates of SARS-CoV-2 attack (point prevalence) and cumulative attack, underdetection, notification and household secondary attack, all of which were expressed as percentages.

The attack rate was calculated by dividing the number of SARS-CoV-2-positive participants identified through the study by the number of participants who had a specimen collected for SARS-CoV-2 testing. The cumulative attack rate was calculated by dividing the total number of all SARS-CoV-2-positive participants (including participants with verified positive test results from the previous 2 weeks who did not have a specimen collected in the study) by the total number of participants with known test results. The underdetection rate was defined as the proportion of SARS-CoV-2-positive participants who did not self-report having a recent positive SARS-CoV-2 test result or were not identified in the notification database. The undernotification rate was defined as the proportion of SARS-CoV-2-positive participants – both those detected during the study and those who self-reported testing positive in the previous 2 weeks – who had a corresponding notification. The household secondary attack rate was defined as the number of secondary cases within a household with at least one case divided by the total number of participants within that household.

Secondary outcomes included symptoms reported during the previous 4 weeks, the number of participants who had a specimen tested for SARS-CoV-2 since the start of community transmission, the secondary household attack rate in households and associations with SARS-CoV-2 positivity. Univariate associations with SARS-CoV-2 positivity assessed included sex, age, vaccination status and prevention measures adhered to (coughing into elbow, handwashing, mask wearing, maintaining physical distance and staying home). Data were analysed using Microsoft Excel and Stata version 17 (StataCorp 2021; Stata Statistical Software, Release 17; College Station, TX, USA).

### Ethical considerations

Ethical approval was obtained from the Chair of the Vanuatu Ministry of Health Research and Ethics Committee. Written consent was obtained from all adults aged over 18 years. For those aged under 18 years, parental or caregiver written consent was obtained. Participants were informed of their results via a phone call and information on isolation was provided as per existing Ministry of Health protocols. Cases were advised of the symptoms of severe disease and to call an ambulance or travel to their closest health facility if they developed severe disease. Strict infection control procedures were in place during the survey process, including routine testing of fieldwork staff, wearing of appropriate personal protective equipment, outdoor data collection and maintenance of optimal physical distance at all times while collecting specimens.

## Results

### Participation rate

The cross-sectional survey was conducted over 3 days on 7, 8 and 14 April 2022; data collection was delayed because of the time required to ensure local authority and chief approvals and because of a funeral in one area. In total, 363 people were eligible across the two study sites and 252 people participated (69.4% participation rate). Sixteen empty houses were not included in the denominator.

### Description of participants

Most participants were aged 18–34 years (range 0–81 years, average 32 years), and 60% were female (**Table 1**). Over half (66.3%) of adult participants were fully or partially vaccinated. There was no statistical difference between study sites for age or sex, but self-reported receipt of a COVID-19 booster shot and having a previous positive SARS-CoV-2 test differed between the two groups (*P* < 0.05). A total of 84 households participated, with a mean of 7.1 people per house (range 1–13 people); household size did not differ significantly between study sites.

**Table 1:** Description of participants in two administrative areas of Port Vila, Vanuatu, April 2022

Characteristic	Study site 1		Study site 2		Total		*P*
	n	%	n	%	n	%	
**Total**	127	50.4	125	49.6	252	100	
**Age (years)**							
< 5 years	6	4.7	10	8.0	16	6.3	> 0.05
5–17 years	19	15.0	24	19.2	43	17.1	
18–34 years	53	41.7	35	28.0	88	34.9	
35–54 years	27	21.3	33	26.4	60	23.8	
≥ 55 years	21	16.5	21	16.8	42	16.7	
Unknown	1	0.8	2	1.6	3	1.2	
**Sex**							
Male	51	40.2	48	38.4	99	39.3	> 0.05
Female	75	59.1	77	61.6	152	60.3	
Unknown	1	0.8	0	0.0	1	0.4	
**Vaccination status**							
Fully or partially vaccinated	84	66.1	83	66.4	167	66.3	**0.001**
Not vaccinated	43	33.9	40	32.0	83	32.9	
Missing	0	0.0	2	1.6	2	0.8	
**Description of households**							
Number of households	38	45	46	55	84	100	
Average household size (range)	7.1	(3–13)	7.2	(1–12)	7.1	(1–13)	> 0.05

Bold *P*-values are statistically significant.

### Primary outcomes

A total of 175 participants had a specimen collected in this study (69% of all participants), with 89 having a positive SARS-CoV-2 test result, giving an attack rate of 50.9% (95% confidence interval [CI]: 43.2–58.5%; **Table 2**). The cumulative attack rate was 55.3% (95% CI: 47.9–62.6%), because 104 participants were positive for SARS-CoV-2 infection, including 15 participants who had positive results notified to the NSRERU but who did not have a specimen collected in the study. Among the 104 SARS-CoV-2-positive participants, 15 self-reported having a positive SARS-CoV-2 test before the study, giving an underdetection rate of 85.6% (95% CI: 77.3–91.7%).

**Table 2:** SARS-CoV-2 positivity, underdetection and undernotification among participants from two administrative areas of Port Vila, Vanuatu, April 2022

Outcome	*n*	%	95% CI
**SARS-CoV-2 positivity**			
Attack rate (point prevalence)	89	50.9^a^	43.2–58.5
Cumulative attack rate	104^b^	55.3^c^	47.9–62.6
**SARS-CoV-2 underdetection**			
Number of positive participants that were not detected before the study (self-reported and verified)	89	85.6^d^	77.3–91.7
**SARS-CoV-2 notification rate**			
Participants self-reporting previous positive test result	23	9.2^e^	3.2–15.1
Participants self-reporting previous positive test result with corresponding notification to surveillance unit	15	65.2^f^	49.4–81.0
**SARS-CoV-2 testing**			
Participants reporting having had a specimen tested for SARS-CoV-2 during the previous month	31	12.3^e^	5.4–19.2
Positive participants reporting having had a specimen tested for SARS-CoV-2 during the previous month	22	20.4^d^	11.5–29.2
**Household attack rate**			
Number of households with at least one case	50	59.5^f^	44.4–74.6
Secondary household attack rate	–	47.7^g^	34.2–61.2

CI: confidence interval; SARS-CoV-2: severe acute respiratory syndrome coronavirus 2.

^a^ Denominator includes all 175 participants who had a specimen collected in the study.

^b^ Numerator includes 89 participants detected in this study plus 15 participants with verified previous infection.

^c^ Denominator includes 188 participants with known SARS-CoV-2 test result, excluding those with no testing history.

^d^ Denominator includes all 104 SARS-CoV-2-positive participants.

^e^ Denominator includes all 252 participants.

^f^ Denominator includes all 84 households.

^g^ Rate is only calculated for 50 households with at least one case.

An additional 10 participants who reported having received a previous positive SARS-CoV-2 test result did not have a specimen collected in this study. The 23 participants who self-reported a positive SARS-CoV-2 test result before the study had the test conducted at the hospital (*n* = 10), provincial health clinic (*n* = 4), private clinic (*n* = 4), workplace (*n* = 3) or home (*n* = 2, data not shown). Among these 23 participants, a corresponding notification was identified for 15, giving a notification rate of 65.2% (95% CI: 42.7–83.6%).

Over half of the 84 households (*n* = 50, 59.5%) had at least one SARS-CoV-2 case, giving a secondary household attack rate of 47.7% (95% CI: 33.2–62.2%) (**Table 2**).

### Secondary outcomes

**Fig. 1:**
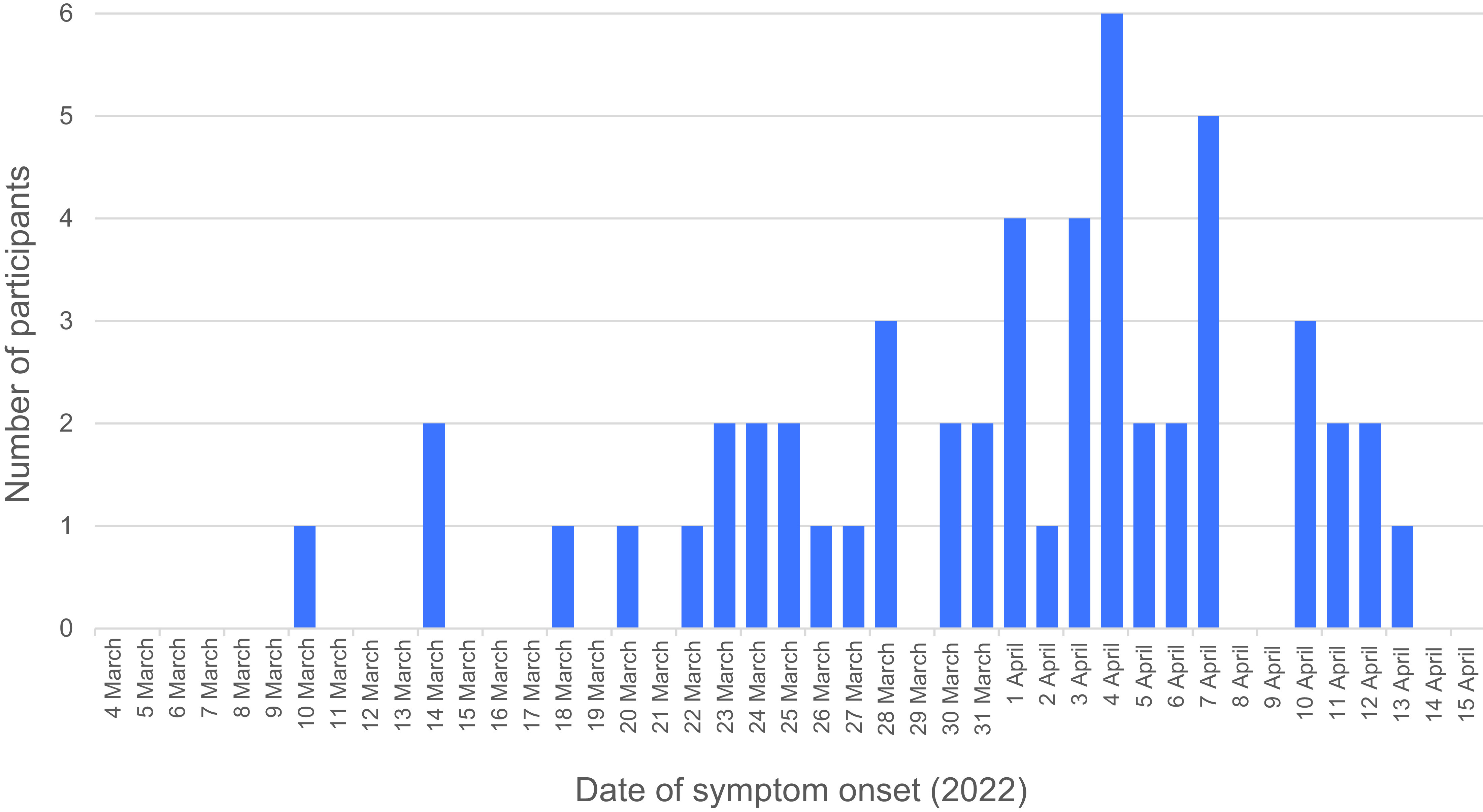
Epidemic curve of symptom onset of SARS-CoV-2-positive participants in two administrative areas of Port Vila, Vanuatu, March–April 2022

Most participants who were positive for SARS-CoV-2 reported recent COVID-19 symptoms (*n* = 83, 80.6%, 95% CI: 63.0–98.2%). **Fig. 1** shows the epidemic curve of symptom onset in such participants. A total of 31 participants (12.3%, 95% CI: 8.5–17.0%) reported having a specimen collected for SARS-CoV-2 testing within the previous month. Among participants positive for SARS-CoV-2, 22 (20.1%, 95% CI: 13.6–30.0%) reported having a specimen collected for SARS-CoV-2 testing in the previous month (**Table 2**).

[**insert** Figure 1]

In univariate analysis, the odds of SARS-CoV-2 infection were significantly higher for participants who reported wearing a mask sometimes or never compared to always (odds ratio [OR]: 5.21, 95% CI: 1.47–18.45), or maintaining physical distancing sometimes or never compared to always (OR: 1.83, 95% CI: 1.01–3.36) (**Table 3**).

**Table 3:** Associations with SARS-CoV-2 positivity in two administrative areas of Port Vila, Vanuatu, April 2022

Predictors of SARS-CoV-2 positivity	Odds ratio	*P*	95% CI
**Demographics**			
Male (ref. female)	0.96	> 0.05	0.53–1.72
Age (ref. each additional year of age)	0.99	> 0.05	0.98–1.01
Not vaccinated (ref. any vaccination)	1.22	> 0.05	0.67–2.27
**Prevention measures**			
Coughed into elbow sometimes or never (ref. Always)	1.72	0.07	0.96–3.07
Handwashing sometimes or never (ref. Always)	1.77	0.101	0.89–3.53
Wore a mask sometimes or never (ref. Always)	5.21	** < 0.01**	1.47–18.45
Maintained physical distancing sometimes or never (ref. Always)	1.83	** < 0.05**	1.01–3.36
Stayed home except for essential movements (ref. Always)	1.08	0.45	0.46–2.55

CI: confidence interval; SARS-CoV-2: severe acute respiratory syndrome coronavirus 2.

Bold *P*-values are statistically significant.

## Discussion

This study is one of the first to publish evidence for the rapid community transmission of SARS-CoV-2 in a Pacific island country. It provides novel evidence that 52% of the study population were SARS-CoV-2-positive within a few weeks of the first community case being identified in Vanuatu. This, and a high secondary attack rate, reflected a short incubation period and serial interval. The underdetection rate of 91.5% suggests that, at the time of the study, about 9 in 10 cases of SARS-CoV-2 had not been diagnosed. Optimistically, the results suggest that over half of detected cases had been notified to the NSRERU.

The high rates of underdetection suggest insufficient testing. WHO recommends minimizing the test positivity rate to less than 5% to indicate comprehensive surveillance of suspected cases; (*12*) however, the test positivity for the study was high at 52%. The reasons for these low testing rates are multifaceted and involve structural, health system and psychosocial factors. Private car ownership is low in Vanuatu, with most of the population using an informal system of privately owned minibuses. Restricted bus services and road barriers prevented movement of people into and within Port Vila; also, loss of income during the COVID-19 pandemic reduced capacity to pay for bus fares. At the time of the study, the main location with free community-based testing was in the grounds of Vila Central Hospital. Government policy at the time was for people testing positive to be immediately taken in buses to a community isolation centre. Anecdotally, there was considerable fear of testing in Port Vila because of this requirement. There was also hesitancy towards testing owing to caregiver and family responsibilities. Further community-based research may be warranted to fully understand barriers to testing, because these are critical for pandemic preparedness and response activities.

The initial community cases in Port Vila were of the BA.1 and BA.2 sublineage of the Omicron variant. Compared with the Delta variant, the Omicron variant had higher transmissibility, (*13*) a shorter incubation period and serial interval, (*14*) a higher rate of asymptomatic infection (*15*) and a lower rate of severe infection. (*16*) These factors intrinsic to the Omicron variant are likely to have driven the high attack rate and high underdetection rate in Port Vila, in addition to sociocultural and housing factors. Relatively few studies have been conducted to investigate underdetection of the Omicron sublineage; studies conducted in France (*17*) and South Africa (*18*) reported similar underdetection rates of 90–95%. The level of underdetection reported here demonstrates the importance of using a range of surveillance data when interpreting case-based surveillance data such as the case-fatality rate or hospitalization rate.

The findings suggest that health-care workers were diligent in notification requirements during the study period. Until 2022, Vanuatu had a paper-based notification system whereby medical officers submitted notifications to surveillance officers via phone, e-mail or in person. An informal assessment among health-care workers in 2021 revealed poor knowledge about notification requirements and processes for COVID-19. Therefore, the NSRERU conducted activities such as rapid development and roll-out of an electronic notification form and brief training of health-care workers on notification processes to undertake once a case was identified. The notification rate reported here is a positive reflection of these surveillance-strengthening activities; however, further work is required to ensure notification of all notifiable diseases beyond SARS-CoV-2, to facilitate case investigation and response.

The reported household secondary attack rate of 48% was high compared with similar reports internationally; an updated systematic review in March 2022 reported a pooled household secondary attack rate of 43% for the Omicron variant. (*19*) The secondary household attack rate reported is likely to underestimate the true household attack rate because some households may have been experiencing within-household transmission at the time of the study; therefore, some secondary cases may not yet have occurred. Household attack rates in other Pacific island countries are not known but are expected to be similarly high owing to a range of social, cultural and environmental factors (e.g. large household sizes due to extended families sharing housing, cooking, water and sanitation facilities across many families, low health literacy and higher density housing). (*20*)

Our analysis suggests that consistent mask wearing and physical distancing were protective against infection, and that mask wearing was the most protective public health and social measure (PHSM) identified. This is consistent with international evidence (*21*, *22*) and is the first evidence for effectiveness of PHSMs in community settings based in a Pacific island country. The Ministry of Health messaging of wearing a mask and physical distancing was therefore warranted and successful in Vanuatu, and should be retained for future respiratory virus outbreaks.

The findings of this study may be considered generalizable across Port Vila and to Vanuatu’s second small urban centre of Luganville in the north of the country, which had similar housing, commercial and government hubs, transportation and road access and implementation of COVID-19 containment policies such as stay-at-home orders. The age and sex structure of the sample was broadly similar to that reported for Port Vila in the 2020 census, although the average household size reported here was higher (7.1 compared with 4.7 people per household). (*23*) Similar definitions of a household were used in this study and the 2020 Vanuatu census, and thus the higher household size reported here may be due to households temporarily living together during the stay-at-home order period. The results may also be considered generalizable to small urban centres in other Pacific island countries, but are less generalizable to rural areas and small islands that do not have government or commercial hubs or road access, and where communication on containment policies were not easily delivered owing to limited communication infrastructure. In these settings, transmission and secondary attack rates may have been greater; however, there are insufficient data to demonstrate this.

Some limitations should be considered. Misclassification of true cases not detected due to insufficient viral load may have occurred due to older infection or new infection; however, RT–PCR has high sensitivity with a long period of detection (up to 90 days). (*11*) Participants who reported having a positive test but for whom a notification was not identified may be underestimated owing to the common practice of both formal names and nicknames in Vanuatu. We attempted to minimize this bias by using a recently introduced national identification number; however, many participants did not report their number in interviews. Recent intra-household transmission may not have been captured, underestimating household secondary attack rates. In addition, biases may have been associated with self-reported health-care seeking behaviours and recall; however, the interviewers were trained to ensure accurate recall and reporting. Usual residents may have been excluded owing to not being home on the day of fieldwork; however, this is anticipated to be low because of the stay-at-home orders in effect and the use of road blockages. Finally, and as described above, the results cannot be considered generalizable to the whole of Vanuatu.

Despite these limitations, this study provides important evidence for the rapid spread of novel respiratory diseases in Vanuatu and the findings are potentially useful for pandemic preparedness and response across the Pacific, particularly for small urban areas. Surveillance systems are fundamentally important to the monitoring and control of infectious diseases; however, a high level of underdetection and undernotification may be expected and should be anticipated when planning for future outbreak detection and control activities.
